# Optimization of the extraction process and metabonomics analysis of uric acid-reducing active substances from *Gymnadenia* R.Br. and its protective effect on hyperuricemia zebrafish

**DOI:** 10.3389/fnut.2022.1054294

**Published:** 2022-12-05

**Authors:** Tianrong Chen, Duoji Pubu, Wenhui Zhang, Shengya Meng, Cuicui Yu, Xiaoqing Yin, Jiale Liu, Yuhong Zhang

**Affiliations:** Institute of Food Science and Technology, Tibet Academy of Agricultural and Animal Husbandry Sciences, Lhasa, China

**Keywords:** *Gymnadenia* R.Br., uric acid-reducing active substances, metabolites, antioxidant, alkaloids

## Abstract

**Background:**

As Gymnadenia R.Br. (Gym) has an obvious uric acid-lowering effect, but its specific bioactive substances and mechanism are still unclear. The key metabolites and pathways used by Gym to reduce uric acid (UA) were identify.

**Methods:**

An optimized extraction process for urate-lowering active substances from Gym was firstly been carried out based on the xanthine oxidase (XOD) inhibition model *in vitro*; then, the Ultra-high-performance liquid chromatography and Q-Exactive mass spectrometry (UHPLC-QE-MS) based on non-targeted metabolomics analysis of Traditional Chinese Medicine were performed for comparison of Gym with ethanol concentration of 95% (low extraction rate but high XOD inhibition rate) and 75% (high extraction rate but low XOD inhibition rate), respectively; finally, the protective effect of ethanolic extract of Gym on zebrafish with Hyperuricemia (referred to as HUA zebrafish) was explored.

**Results:**

We found that the inhibition rate of Gym extract with 95% ethanol concentration on XOD was 84.02%, and the extraction rate was 4.32%. Interestingly, when the other conditions were the same, the XOD inhibition rate of the Gym extract with 75% ethanol concentration was 76.84%, and the extraction rate was 14.68%. A total of 539 metabolites were identified, among them, 162 different metabolites were screened, of which 123 were up-regulated and 39 were down-regulated. Besides significantly reducing the contents of UA, BUN, CRE, ROS, MDA, and XOD activity in HUA zebrafish by Gym and acutely reduce the activity of SOD.

**Conclusion:**

Along with the flavonoids, polyphenols, alkaloids, terpenoids, and phenylpropanoids, the ethanolic extract of Gym may be related to reduce the UA level of Gym.

## Introduction

Known as *Gymnadenia conopsea* (L.) R.Br. (Gym, Orchidaceae), it grows widely in temperate and subtropical regions of Asia as well as throughout Europe. Traditionally, its tuber is applied to Traditional Chinese Medicine, Tibetan Medicine, Mongol Medicine, and other medicines to treat numerous symptoms of health problems in China (1, 2). Various research has been carried out on composition analysis to pharmacological analysis. So far, regarding the composition analysis side, hundreds of compounds have been identified, mainly glucosides, dihydrostilbenes, phenanthrenes, and aromatic compounds (3–9). Among them, high-performance liquid chromatography (HPLC) analysis is commonly performed. An HPLC-diode array detection-tandem mass spectrometry method (HPLC-DAD-MSn) was initially established for the analysis of chemical fingerprints of Gym rhizomes and the rapid identification of major compounds (10). Lin et al. (11) developed a sensitive ultra-high performance liquid chromatography (UPLC)-HRMS/MS method for the rapid screening and identification of compositions in bioactive fractions. Forty-six compounds were identified from the ethanolic extract of Gym by extraction ion chromatography (EIC). Besides, a fast and precise system based on the combination of UPLC and Orbitrap MS/MS was established by Wang et al. (7) finally, 91 compounds were identified by using both positive and negative ion modes in Gym tubers for the first time. The bioactive substances and biological efficacy of Gym have attracted extensive attention, and more and more methods have been developed for screening and identification of their metabolites.

Gym and its active constituents possess a wide range of pharmacological properties, including tonification effect (12), anti-oxidant properties (3, 13), immunoregulatory (14), anti-anaphylaxis (15), anti-gastric ulcer (16), sedative, and hypnotic activities (17–21). Liang et al. (22) screened 20 main chemical components of the Gym through network pharmacological methods, with 304 potential anti-hypoxia targets. It was found that the main active components play an anti-hypoxia role by acting on H1F1α, TNF-α, mTOR, and other targets (22). It has been verified that the ethanolic extract of Gym can substantially reduce silica dust-induced lung coefficient in rats, significantly reduce the synthesis of type I and III collagens, and substantially inhibit pulmonary fibrosis in rats (23). lncRNAs expression profiles in mice treated with Gym for high-altitude hypoxia-induced brain injury were examined by Wenhui Zhang et al. using microarray methods. They found significant dysregulation of 126 mRNAs, differential expression of 226 lncRNAs, and 23 circRNAs. The results also revealed that the mRNAs co-expressed with lncRNAs mainly related to stress, inflammatory reaction, and hypoxia, including the H1F1α and PI3K-Akt signaling pathways (24). Additionally, Feng et al. proved that the Gym polysaccharide has a therapeutic effect on ionizing radiation-induced impairment of hematopoietic and antioxidant function in mice (25). This indicates that it is feasible to analyze the role of Gym in antioxidant and biological metabolism, and the pathway research on specific diseases will be more exciting.

Hyperuricemia (HUA) is caused by a disorder of uric acid (UA) metabolism or abnormal purine metabolism. It occurs when the serum UA level is higher than 420 μmol/L (26–28). The prevalence of HUA is increasing rapidly, not only in China but also worldwide (29, 30). The global prevalence of HUA is 5–25%, another common metabolic disease after diabetes (31, 32). As a key enzyme for the production of UA, xanthine oxidase (XOD) can directly catalyze the gradual oxidation of hypoxanthine and xanthine to form UA, and can also convert the rich protein in food into purine to finally form UA. Therefore, the inhibitors of XOD are widely used in the treatment of HUA (33–39). As a purine analog and hypoxanthine isomer, allopurinol (APL) is an important inhibitor of urate reduction (40–42). Yan et al. established an XOD inhibitor screening system by measuring XOD activity *in vitro* using physical and chemical methods (43). Chen et al. established an *in vitro* inhibitory model of XOD to understand the inhibitory properties of quercetin, rutin, and gallic acid and their combination with vitamin C on XOD (44). Park et al. reported that the ethanolic extract of Aster glehni can effectively reduce the XOD activity and serum UA level of HUA rats induced by potassium oxalate (PO) (45). Le et al. (46) found that by inhibiting the mRNA expressions, AST reduced UA synthesis and inhibited XOD and ADA enzyme activities, thereby alleviating HUA (46). Science Gym has a significant effect on lowering UA, and its correlation with the XOD and underlying antioxidant stress protection mechanism is deserved to be explored.

Therefore, whether the ethanolic extract of Gym can reduce the production of UA by inhibiting XOD activity, thereby alleviate HUA. In this work, we will focus on this hypothesis. To this end, specific ethanolic extractions based on different concentrations of Gym should be carried out first to ensure the representativeness of the samples. First, we optimized the extraction process of UA-reducing active substances of Gym and analyzed the metabolomics of the ethanolic extract of Gym by UHPLC-Q-Exactive mass spectrometry (UHPLC-QE-MS). Finally, the protective effect of the ethanolic extract from Gym on HUA zebrafish was explored. Consequently, it can well explore the possible metabolic effect of ethanolic extract of Gym on the reduction of UA and its protective effect on HUA zebrafish.

## Materials and methods

Together with bioinformatics, UHPLC-QE-MS was applied for chemical identification, profiling, and quantitation. UHPLC-QE-MS used a high pressure of more than 1,000 bars and worked at a tolerable flow of up to 5 mL/min. These properties allowed it to analyze slighter particles less than 2.2 mm. Besides the traditional HPLC method, UHPLC-QE-MS detection was an ideal estimating method for pharmaceutical formulations or drugs in bulk and analyzing the metabolites in biological fluids (47). The technology was widely used in the analysis of Traditional Chinese Medicine (48, 49).

### Chemical materials and the sources

Xanthine sodium salt (XSS, CAS:1196-43-6), bovine milk XOD (CAS:9002-17-9), APL (CAS:315-30-6), and formic acid (CAS:67-56-1, MS grade) were provided by Sigma Chemical Co., LTD (Shanghai, China). Methanol (CAS:67-56-1) and acetonitrile (CAS:75-05-8) (all MS grade) were offered by CNW Technologies (Shanghai, China). The 2-chloro-L-phenylalanine (CAS:103616-89-3, MS grade) was bought from Shanghai Hengbai Biotechnology Co., LTD (Shanghai, China). Ethanol absolute was purchased from Chengdu Jinshan Chemical Reagent Co., LTD (Chengdu, China). 10 × PBS buffer (pH 7.4) was offered by Shanghai Thermo Fisher (Shanghai, China). The gym was obtained from the Tibet specialty warehouse naicang supermarket (Tibet, Lhasa, China).

### Establishment of xanthine oxidase inhibition model *in vitro*

According to the study of Chen et al., the XOD inhibition model *in vitro* was established and further optimized (44, 50). As shown in Supplementary Figure 1, when XSS concentration was 100 μM, XOD concentration was 5 U/L, and reaction time was 15 min, the absorbance value of UA remained unchanged and the enzymatic reaction was complete, indicating that the XOD inhibition model was successfully established *in vitro*. The IC_50_ value of APL was 0.773 μg/mL, the 95% confidence interval was 0.449–1.331 μg/mL (Supplementary Figure 2).

### Single-factor experiment design

The single-factor test was set according to Supplementary Table 1. The sample extraction steps were as follows. First, 10 g Gym powder was accurately weighed, and 75% ethanol was added according to the solid–liquid ratio of 1:10, 1:20, 1:30, 1:40, and 1:50. The solutions were sonicated at 500 W at 50°C for 40 min, filtered, and concentrated at 50°C with a rotary evaporator, and the extraction rate was calculated after drying. The solution was stored at room temperature for the next experiments. Optimization experiments were conducted using the one-way controlled variable method, and all experiments were repeated three times (51–56).

### Determination of xanthine oxidase inhibition rate

The concentration of Gym was determined as 4 g/L by pre-experiment to measure the inhibition rate of XOD (Supplementary Figure 3). The Gym samples were extracted under different factors and levels. First, PBS, and XSS were added to the 96-well plates, then XOD (pre-incubated at 37°C in the dark for 30 min to stabilize the enzyme activity) was added. Amounts are shown in Supplementary Table 2. The mixture was evenly mixed, and the reaction was carried out at 37°C for 15 min and finally measured at 295 nm (57–59). All determinations were repeated three times. Inhibition rate = [1-(Ai-Aj)/(A0-A1)] * 100%, where A0, A1, Ai, and Aj represent the absorbance of the negative control experimental group, negative control blank group, sample/positive experimental group, and sample/positive blank group at 295 nm, respectively.

### Orthogonal experiment

The 4-factors and 3-levels orthogonal L_9_ (3^4^) test design was carried out according to Supplementary Table 3. Nine experiments were performed in triplicate based on Section “Determination of xanthine oxidase inhibition rate” (60, 61).

### Ultra-high-performance liquid chromatography and Q-Exactive mass spectrometry analysis

The samples of Gym with 95 and 75% ethanol concentration were extracted and analyzed by LC/MS. Briefly, the Waters UPLC BEH C18 column was used on Agilent 1,290 UPLC system (1.7 μm 2.1*100 mm, Waters) for LC-MS/MS analysis (62–64). The injection volume was 5 μL and the flow rate was 0.4 mL/min. Next, 0.1% formic acid was added to the water (A) and acetonitrile (B) phases (65, 66). The QE-MS and Xcalibur software were applied to acquire the MS and MS/MS data according to the IDA acquisition mode. Corresponding parameters refer to Luo et al. (67, 68). These experiments were carried out by Shanghai Biotree Biotech Co., Ltd. (Shanghai, China).

### Assessment of uric acid-reducing effect

In reference to Zhang et al. (69) and Xiong et al. (70), an XOD model was established with PO combined with xanthine sodium salt (XSS) on Zebrafish. A total of 660 embryos with normal development in 5 dpf were selected and divided randomly into 11 groups. Three parallel lines were set in each group and placed in 12-well plates (*n* = 20/group), respectively, and the volume of each well was 2 mL. Each culture dish was marked with the group and treatment. Except for the blank group, the other 10 groups were treated with PO and XSS, pre-incubated at 28°C for 1 h, and the corresponding dosages of APL and Gym extraction aqueous solution were added. All treatment groups were cultured in a 28°C incubator for 24 h. The contents of UA, Blood Urea Nitrogen (BUN), Creatinine (CRE), Malondialdehyde (MDA), and the enzyme activity of XOD and Superoxide Dismutase (SOD) were measured according to the kit’s instructions. The content of Reactive Oxygen Species (ROS) in 48 h was also determined by model reference in the meantime (71–75).

### Data statistics and analysis

The GraphPad Prism software (version 6.0, GraphPad Software Inc., San Diego, CA, USA) was used to conduct one-way ANOVA analysis through a *post-hoc* Dunnet *T*-test for statistical analysis. Data were expressed as mean ± standard deviation (SD). In all statistical comparisons, *P <* 0.05 was considered significant, and *P <* 0.01 was extremely significant. Besides, principal component analysis (PCA) and orthogonal partial least squares discriminant analysis (OPLS-DA) was conducted to process the metabolomics analysis using SIMCA software (V14.1, MKS Data Analytics Solutions, Umea, Sweden). This was of importance when Variable Importance in the Projection (VIP) *>* 1 while *P*-value *<* 0.05.

## Results

### Single-factor experimental analysis

#### Effect of liquid–solid ratio on xanthine oxidase inhibition rate

**FIGURE 1:**
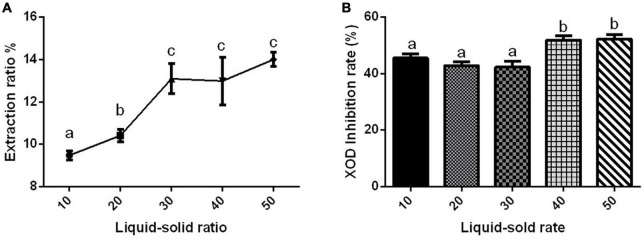
The effect of liquid–solid ratio. **(A)** The effect of liquid–solid ratio on the extraction rate of ethanolic extract of Gym. **(B)** The effect of liquid–solid ratio on xanthine oxidase (XOD) inhibition rate. The different letters indicate significant difference (*P* < 0.05).

The extraction rate of Gym ethanolic extract was greatly affected by different liquid–solid ratios, and they were positively correlated (Figure 1A). However, the difference in the XOD inhibition rate affected by different liquid–solid ratios was not obvious (Figure 1B). The liquid–solid ratio of 1:40 and 1:50 significantly inhibited XOD by about 50%, but with no significant difference between the two groups. Therefore, three liquid–solid ratios of 1:10, 1:20, and 1:40 were selected as the three levels of the orthogonal experiment.

#### Effect of ethanol concentration on xanthine oxidase inhibition rate

**FIGURE 2:**
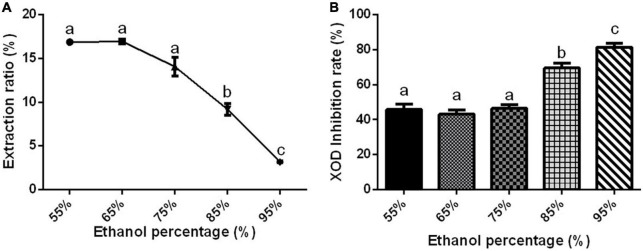
The effect of ethanol concentration. **(A)** The effect of ethanol concentration on the extraction rate of ethanolic extract of Gym. **(B)** The effect of ethanol concentration on xanthine oxidase (XOD) inhibition rate. The different letters indicate significant difference (*P* < 0.05).

Substances have different solubility and content in different ethanol concentrations (76, 77). The extraction rate of Gym decreased significantly with the increase in ethanol concentration (Figure 2A). Interestingly, the XOD inhibition rate remained insignificant, around 40%, when the ethanol concentration ranged from 55 to 75%. But the XOD inhibition rate increased significantly with the increase of ethanol concentration to more than 75% (Figure 2B). When the ethanol concentration was raised to 95%, the XOD inhibition rate reached the highest, up to 80%. Therefore, considering the factors such as extraction rate and practical application, two ethanol concentrations of 95 and 75% were selected for the next optimization experiment.

#### Effect of ultrasonic power, extraction time, and extraction temperature on xanthine oxidase inhibition rate

**FIGURE 3:**
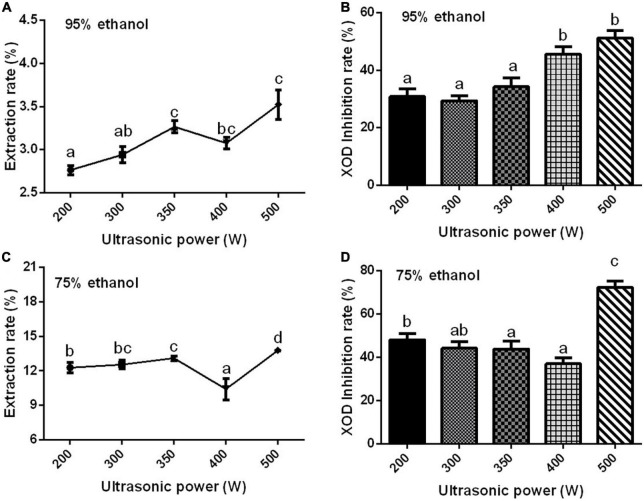
The effect of ethanol concentration. **(A,C)** The effect of ultrasonic power on the extraction rate of ethanolic extract of Gym [**(A)** 95% and **(C)** 75%]. **(B,D)** The effect of ultrasonic power on XOD inhibition rate [**(B)** 95% and **(D)** 75%]. The different letters indicate significant difference (*P* < 0.05).

Ultrasonic power is one of the main factors affecting the extraction rate of effective components. If the power is not selected properly, the components contained in the sample will be extracted incompletely (78–80). With the increase in ultrasonic power, the extraction rate of the Gym increased first, then decreased, and then increased significantly (Figures 3A,C). When the ultrasonic power was 500 W, the XOD inhibition rates of the two ethanolic extracts reached the maximum (Figures 3B,D). Therefore, according to Figure 3, three ultrasonic powers of 200, 350, and 500 W were selected as the three levels of the orthogonal experiment. The effect of extraction time and temperature on XOD inhibition rate are presented in Supplementary Figures 4, 5. So based on the results, three times of 20, 40, and 80 min were selected as the three levels of the orthogonal experiment, and 70°C was determined as the optimum extraction temperature.

### Analysis of orthogonal experimental results

The extraction rate and XOD inhibition rate of the two ethanolic extracts of Gym from the orthogonal experiment were listed in Supplementary Tables 4, 5. When the ultrasonic power was 500 W, the liquid–solid ratio was 1:40, extraction time was 80 min, the XOD inhibition rates of Gym with 95 and 75% ethanol concentration were 84.02 and 76.84%, respectively, and the extraction rates were 4.32 and 14.68%, respectively. Namely, the extraction conditions were a combination of experimental factors called A3B3C3 (Supplementary Tables 6–9 for the results of the analysis of variance). Under these conditions, the XOD inhibitory activity of the two ethanolic extracts was verified *in vitro* (Supplementary Figure 6). This demonstrated that the two ethanolic extracts significantly increased the inhibition rate of XOD and the difference was statistically significant.

### Ultra-high-performance liquid chromatography and Q-Exactive mass spectrometry detection results

#### Multivariate statistical analysis

We want to know why the XOD inhibition rate of 95% ethanolic extract of Gym was significantly higher than that of 75% ethanolic extract *in vitro*. In comparison, the 95% ethanolic extract of Gym was far lower than that of the 75% ethanolic extract. What are the differences and common substances between the two ethanolic extracts of different concentrations? Therefore, we detected the metabolomics of UA reducing effectiveness for 95 and 75% ethanolic extracts of Gym after process optimization by using an advanced UHPLC-QE-MS method. First, we obtained the total ion current (TIC) of QC and 95 and 75% ethanolic extract of Gym under positive and negative ions (Supplementary Figures 7–9). The experimental results showed that the sample quality, experimental method, and system stability are convincing, indicating reliable test results.

**FIGURE 4:**
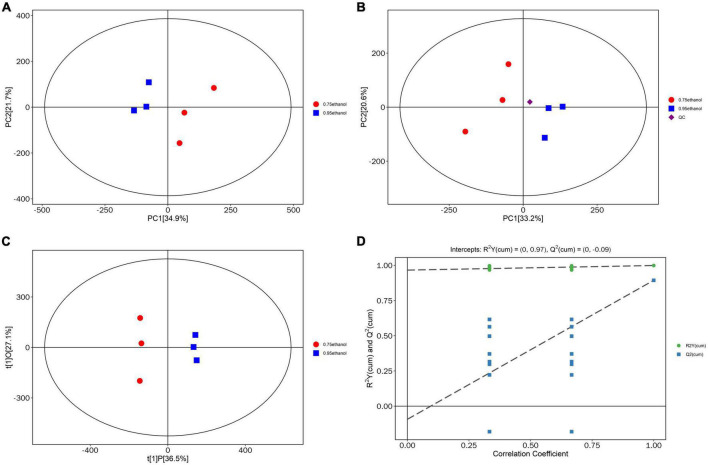
Principal component analysis (PCA) score plot. **(A)** Score scatter plot of PCA model for group 95% ethanol vs. 75% ethanol. **(B)** Score scatter plot for PCA model total with QC. **(C)** Score scatter plot of OPLS-DA model for group 95% ethanol versus 75% ethanol. **(D)** Permutation test of OPLS-DA model for group 95% ethanol versus 75% ethanol.

After data correction, the PCA and OPLS-DA models were applied to Gym extract samples with different ethanol concentrations. PCA reflects the overall variability within and between samples and reveals distribution trends and discrete dispersion points (81–83). Estimates of sample scores in the plane containing the first and second principal components (PC1 and PC2) are spatial coordinates. Intuitively, this indicates the similarity or difference between samples (84–87). Furthermore, the OPLS-DA model implements supervised classification. It can distinguish between two or more groups using multivariate data (88–90). The PCA scores plotted in this study were all within the confidence intervals of 95% (Figure 4A). The samples of ethanolic extract from Gym with the proportion of 95 and 75% were separated and specifically identified by PC1. The tight clustering of QC samples was observed in the middle of the three groups (Figure 4B), suggesting that the experiment was reproducible and stable. It also can be seen from Figure 4C that the OPLS-DA score plots showed a high distinction among sample groups, which were all within the 95% confidence interval. In addition, excellent model parameters [*R*^2^Y (cum) = (0, 0.95), Q^2^ (cum) = (0, −0.09)] were detected in our experiment. Moreover, the OPLS-DA model was not overfitted according to the cross-validation and response permutation test (RPT) (Figure 4D). Therefore, the OPLS-DA model was valid and performed well. This reveals that it can be used to investigate metabolic differences among different concentrations of Gym ethanolic extracts.

### Overview of the metabolites

**FIGURE 5:**
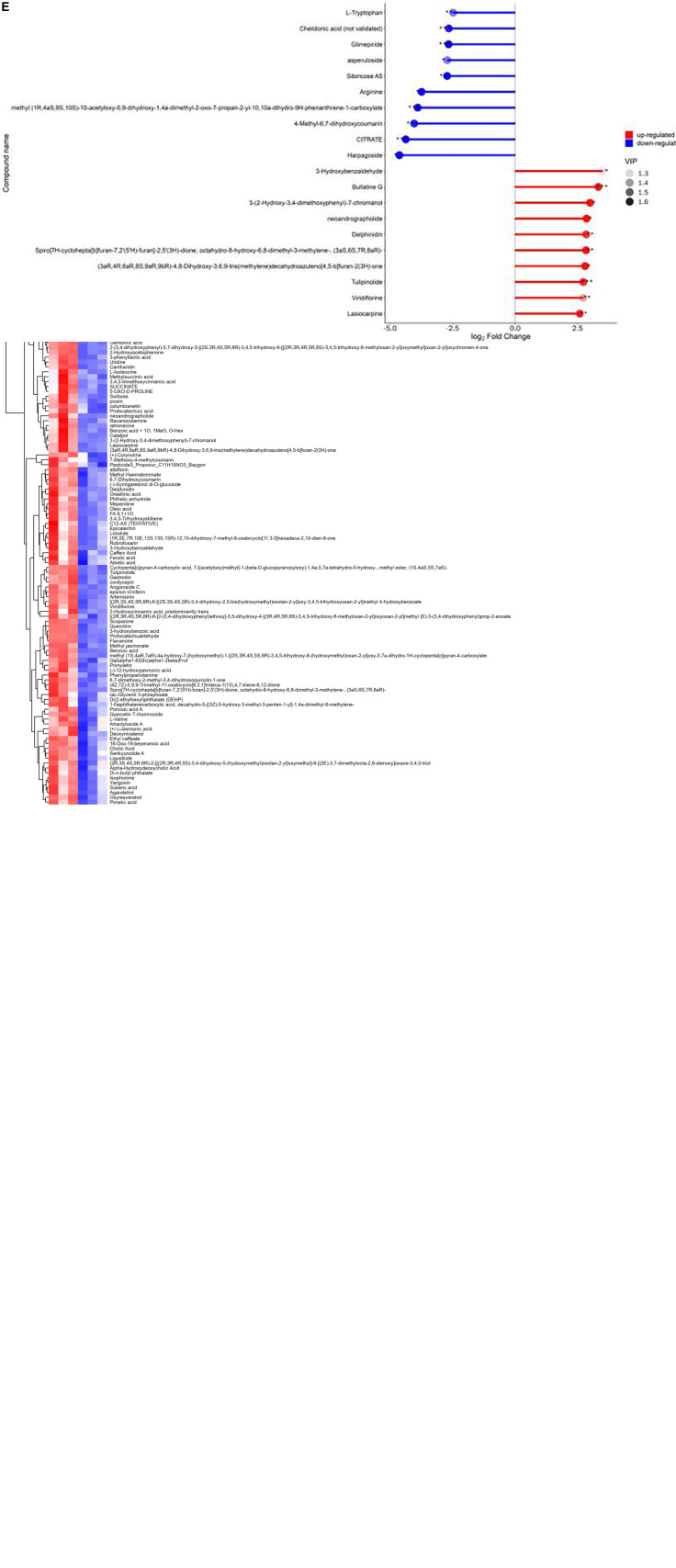
**(A)** Classification of the 539 metabolites detected in ethanolic extract of Gym. **(B)** Classification of the 162 differential metabolites. **(C)** Volcano plot for 95% ethanol versus 75% ethanol group. **(D)** Heat map of hierarchical clustering analysis for 95% ethanol versus 75% ethanol group. **(E)** Matchstick analysis for 95% ethanol versus 75% ethanol group.

A total of 539 molecules comprising 13 classes were identified in 6 ethanolic extract samples of Gym, which included 129 terpenoids, 69 alkaloids, 57 polyphenols, 48 flavonoids, 3 quinones, 36 phenylpropanoid, 24 organic acids, 16 amino acids, 13 sugar and alcohol, 42 lipids and aromatics, 12 coumarins and lignans, 11 carboxylic acid and organic oxygen, and 79 miscellaneous (Figure 5A). The VIP value is often performed to assess the impact strength and interpretability of the inter-group expression pattern as an important parameter in OPLS-DA analysis. The higher the VIP value, the greater the contribution of the variable to the grouping. In essence, metabolites with VIP values greater than 1 are considered differential metabolites (91). Furthermore, *t*-test is required to examine the characteristics of metabolites among groups. And the *P*-value is usually used to evaluate the likelihood of differences between groups (92). When the VIP value is greater than 1 and the *P*-value is less than 0.05, it can be used as the condition for determining potential biomarkers. Accordingly, 162 differential metabolites were screened, of which 123 were upregulated, such as scoparone, artemisinin, methyl jasmonate, benzoic acid, racanisodamine, p-hydroxybenzaldehyde phenylpropanolamine, cholic acid, meperidine, ligustilide, and 3,4,5-trihydroxystilbene. Meanwhile, 39 metabolites showed a downregulation trend, such as paracetamol, L-Proline, heptadecanoic acid, parishin E, cinnamaldehyde, cinnamamide, notopterol, and D-Gluconic acid (Figure 5C and Supplementary Table 10).

These differentiated metabolites have 13 species, including 29 polyphenols, 28 flavonoids, 17 phenylpropanoids, 16 alkaloids, 13 terpenoids, 11 lipids, 7 amino acid derivatives, 6 benzenes and derivatives, 4 organic and aromatics, 5 organic acids and derivatives, 4 coumarins and derivatives, 3 carbohydrates, and 19 miscellaneous (Figure 5B). Meanwhile, hierarchical cluster analysis (HCA) also proclaimed that different ethanolic extracts of Gym had different intensities of different metabolites (Figure 5D). Furthermore, the top 10 elevated and reduced metabolites are shown in Figure 5E. The elevated metabolites include Lasiocarpine, Viridiflorine, Tulipinolide, Delphinidin, Neoandrographolide, Bullatine G, and 3-Hydroxybenzaldehyd. And the top 10 reduced metabolites, including Harpagoside, CITRATE, 4-Methyl-6,7-dihydroxycoumarin, Arginine, Sibiricose A5, Asperuloside, Chelidonic acid (not validated), and L-Tryptophan.

### Kyoto encyclopedia of genes and genomes enrichment analysis

**FIGURE 6:**
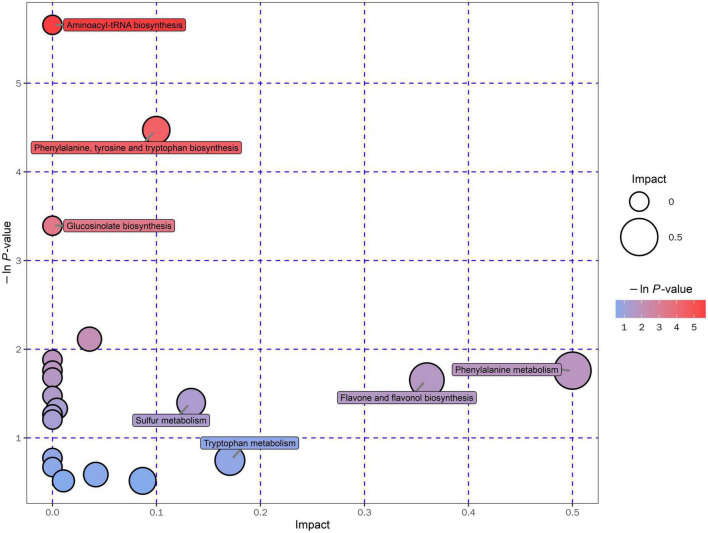
Pathway analysis for 95% ethanol versus 75% ethanol group.

In the present study, the metabolic pathway of differential metabolites was enriched and analyzed according to the KEGG database (Kyoto Encyclopedia of Genes and Genomes)^1^, which is one of the most commonly used biological information databases in the world (93). About 20 metabolic pathways were enriched and presented in an interactive visualization (Supplementary Table 11). The results illustrated that the UA reduction in Gym ethanolic extract mainly involved aminoacyl-tRNA biosynthesis pathway, phenylalanine, tyrosine and tryptophan biosynthesis pathway, glucosinolate biosynthesis pathway, and phenylalanine metabolism pathway (Figure 6).

### The results of uric acid-lowering effect

**FIGURE 7:**
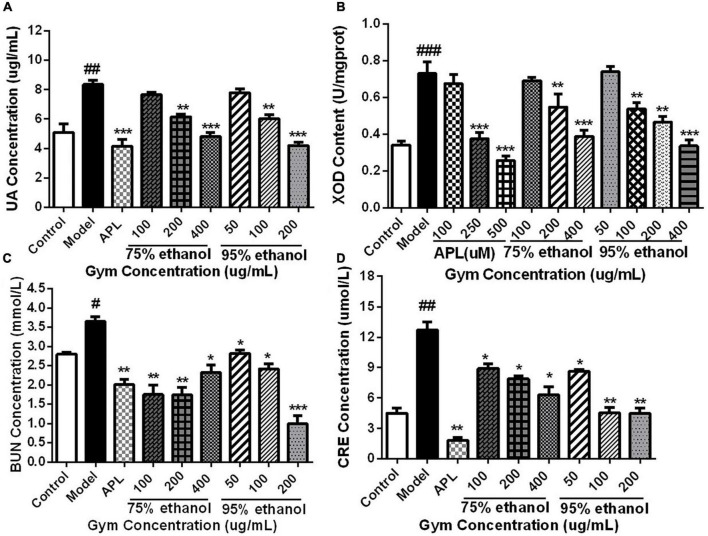
The effects of ethanolic extracts of Gym on UA-related indexes of HUA zebrafish. **(A)** The effect of ethanolic extracts of Gym on UA content. **(B)** The effect of ethanolic extracts of Gym on XOD content. **(C)** The effect of ethanolic extracts of Gym on the content of BUN. **(D)** The effects of ethanolic extracts of Gym on CRE content. *P < 0.05, **P < 0.01, ***P < 0.001. ^#^*P* < 0.05, ^##^*P* < 0.01, ^###^*P* < 0.001.

Based on the HUA zebrafish model, the UA-lowering effect of the Gym ethanolic extracts of two different ethanol degree groups was evaluated. Correspondingly, the results are presented in Figure 7. The UA content and the enzyme activity of XOD were substantially increased in the model group. While decreased significantly in the APL group, the middle, and the high-concentration groups of the two ethanolic extracts (Figures 7A,B). By comparison, the contents of BUN and CRE were also markedly elevated in the model group. However, the two ethanolic extracts of Gym could significantly reduce the contents of BUN and CRE at different concentrations (Figures 7C,D).

**FIGURE 8:**
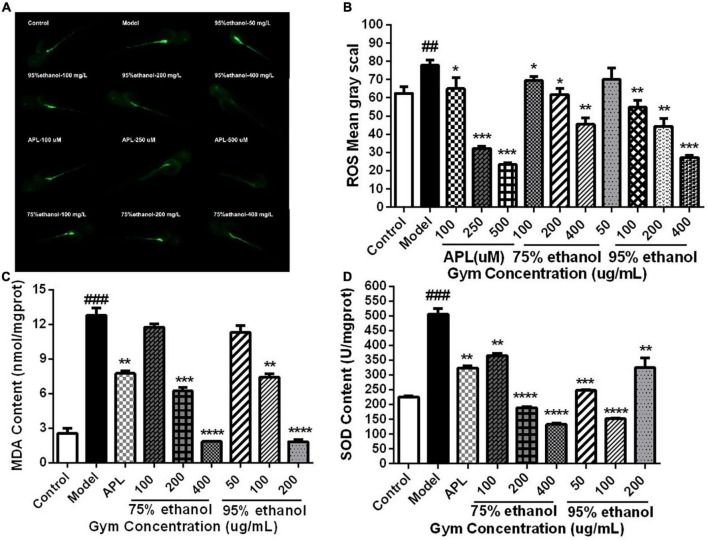
The effects of ethanolic extracts of Gym on oxidative stress-related indexes of HUA zebrafish. **(A)** Fluorescence diagram of ROS of ethanolic extract of Gym. **(B)** Grayscale diagram of ROS content of ethanol extracts of Gym. **(C)** The effect of ethanolic extracts of Gym on MDA content. **(D)** The effects of ethanolic extracts of Gym on SOD content. *P < 0.05, **P < 0.01, ***P < 0.001. ^#^*P* < 0.05, ^##^*P* < 0.01, ^###^*P* < 0.001.

Additionally, we also measured the ROS content of 2 dpf zebrafish embryos stripped of the membrane after 1 h of administration and the contents of MDA and SOD in zebrafish at 5 dpf. It can be seen that the levels of ROS and MDA were substantially increased in the model group. Nevertheless, they could be significantly reduced by APL and different dosages of two ethanolic extracts of Gym groups (Figures 8A–C). However, by contrast, it was found that the activity of SOD increased sharply in the model group, while decreasing significantly in different concentrations of the two ethanolic extracts of Gym groups (Figure 8D). This may be related to the initiation of the antioxidant stress protection mechanism.

## Discussion

The abnormal activity of liver xanthine oxidase or the decreased excretion of UA will lead to an increase in UA accumulation, which is the main cause of HUA (94–96). Therefore, regulating the contents of related enzymes and the level of related UA anion transporters is an important entry point to prevent and alleviate HUA. As a key enzyme for UA production, it can reduce the serum UA level by inhibiting XOD activity, thereby relieving HUA.

In our study, through the XOD inhibition model *in vitro*, the extraction process of UA-reducing active substances in ethanolic extract of the Gym was optimized. When the ethanol concentration was 95%, the ratio of solid to liquid was 1:40, the ultrasonic power was 500 W, the extract time was 80 min, the ultrasonic temperature was 70°C, the inhibition rate of ethanolic extracts of Gym on XOD was 84.02%, and the extraction rate was 4.32%. Interestingly, we found that when the ethanol concentration was 75%, and other conditions were the same, the inhibition rate of Gym on XOD was 76.84% and the extraction rate was 14.68%. In other words, the extraction rate of 75% ethanol is much higher than that of 95% ethanol, and the inhibition rate of 95% ethanolic extract on XOD is significantly higher than that of 75% ethanolic extract.

To clarify this phenomenon, we further analyzed the metabonomics of two ethanolic extracts of Gym based on UHPLC-QE-MS. A total of 539 metabolites were detected, including 129 terpenoids, 69 alkaloids, 57 polyphenols, 48 flavonoids, 3 quinones, 36 phenylpropanoid, 24 organic acids, 16 amino acids, 13 sugar and alcohol, 42 lipids and aromatics, 12 coumarins and lignans, 11 carboxylic acid and organic oxygen, and 79 miscellaneous. Of the 162 different metabolites screened, 123 were upregulated, such as Scopoletin, Artemisinin, Methyl jasmonate, Benzoic acid, Racanisodamine, P-hydroxybenzaldehyde Phenylpropanolamine, Cholic acid, Meperidine, Ligustilide, and 3,4,5-trihydroxystyrene. Meanwhile, 39 metabolites showed a downward trend, such as Paracetamol, L-proline, Heptadecanoic acid, Paregoricin E, Cinnamaldehyde, Cinnamamide, Nortriptyline, and D-gluconic acid. These differential metabolites were classified into 13 species, including 29 polyphenols, 28 flavonoids, 17 phenylpropanoids, 16 alkaloids, 13 terpenoids, 11 lipids, 7 amino acid derivatives, 6 benzenes and derivatives, 4 organic and aromatics, 5 organic acids and derivatives, 4 coumarins and derivatives, 3 carbohydrates, and 19 miscellaneous. There are also various metabolite pathways highlighted in the metabolite pathway enrichment analysis. They are the aminoacyl-tRNA biosynthesis pathway, phenylalanine, tyrosine and tryptophan biosynthesis pathway, glucosinolate biosynthesis pathway, and phenylalanine metabolism pathway. The study of Yao et al. (97) also showed that 33 metabolites were closely related to the improvement of HUA induced by PO by dioscin. Among the 33 metabolites, 5 were lipids. These metabolites were mainly metabolized with arginine and proline; purine metabolism; tyrosine, tryptophan and phenylalanine metabolism; Citric acid cycle; serine, glycine, and threonine metabolism; leucine, valine, and isoleucine metabolism; and glycerol phospholipid metabolism (97–99). The multiple metabolic pathways in this study are consistent with their studies. It is suggested that those pathways are deserved to be studied in future to demonstrate the mechanism of HUA and evaluate the efficiency of treatment.

*In vivo*, UA is proven to be the final product of human purine metabolism. Adenosine is oxidized to inosine by adenosine deaminase, which is further broken down to hypoxanthine, then converted to xanthine by XO, and xanthine is then converted to UA by XO. The whole process is catalyzed by xanthine oxidase (100, 101). The change in renal function can be reflected in the level of BUN. When the BUN level is increased, it may indicate impaired renal function, decreased glomerular filtration rate, and increased CRE level (102). To evaluate the hypouricemic effect of the two Gym ethanolic extracts, we further constructed an acute HUA zebrafish model by combining PO and XSS. The experiment showed that XOD activity, UA, BUN, and CRE content in the positive drug group and the ethanolic extract of the Gym group were significantly lower than that of the model group (*P <* 0.05), which was consistent with a study of Zhao et al. (103–107). It can be seen that the ethanolic extract of Gym can inhibit the activity of XOD of HUA zebrafish to a certain extent and then affect the entire UA production pathway, thus reducing the production of UA.

The available evidence reveals that the formation of UA is catalyzed by XOD, which is a purported source of ROS, and the changes in XOD and UA are biomarkers of oxidative stress. Additionally, XOD may provide an important source of nitric oxide (NO) that quenches the injurious effects caused by ROS (108–110). Li et al. have shown that febuxostat can reduce the content of serum UA in rats, inhibit the occurrence and development of inflammation, reduce oxidative stress, and reduce damage to cerebral arteries and blood vessels (111). Cao et al. (112) found that glycyrrhizin flavonol could reduce the level of adenosine-induced cell UA, increase the activities of SOD, CAT, and GSH content of cells after UA induction, reduce the contents of H_2_O_2_ and MDA, and improve the oxidative stress injury of renal tubular epithelial cells caused by high UA. Its mechanism of reducing UA may be related to XO (112). Wu et al. also showed that hirudin might reduce apoptosis of renal tubular cells caused by UA by improving cell antioxidant capacity, alleviating mitochondrial damage, and reducing oxidative stress response (113). The results in the present study showed that compared with the model group, the levels of ROS and MDA (the lipid peroxidation production) in the positive drug group and the ethanolic extract of the Gym group were significantly reduced (*P <* 0.05), while the content of SOD (the antioxidant enzyme) also decreased stress. This suggests that the ethanolic extract of Gym can attenuate the oxidative stress induced by hyper UA by promoting the activity of antioxidant enzymes and inhibiting lipid peroxidation.

In conclusion, the ethanolic extracts of Gym can significantly improve the HUA of zebrafish. On the one hand, it may be related to its reduction of UA production. On the other hand, it may be related to its significant reduction of BUN and CRE levels, reversing the damage of PO to the kidney, thereby promoting UA excretion, and it may also be related to its antioxidant protection by reducing the production of ROS and MDA, reducing the oxidative stress response.

## Conclusion

The ethanolic extract of Gym can effectively improve the HUA of zebrafish. It can reduce the UA, BUN, CRE content, and XOD level of the HUA zebrafish, suggesting that the ethanolic extract of Gym causes a certain inhibitory effect on the XOD activity of the HUA zebrafish, and then affects the entire UA production pathway, thereby reducing the production of UA. In addition, the ethanolic extract of Gym can substantially reduce the levels of ROS and MDA of HUA zebrafish, and could reduce the SOD level, indicating that the ethanolic extract of Gym can alleviate the oxidative stress reaction caused by hyper UA by improving the activity of related antioxidant enzymes and inhibiting lipid peroxidation. The results of metabonomics showed that its effect on reducing UA might be related to its flavonoids, polyphenols, alkaloids, terpenoids, and phenylpropanoids. And aminoacyl-tRNA biosynthesis pathway, phenylalanine, tyrosine, and tryptophan biosynthesis pathway; glucosinolate biosynthesis pathway, phenylalanine, and other metabolic pathways deserve further study.

## Data availability statement

The original contributions presented in this study are included in the article/Supplementary material, further inquiries can be directed to the corresponding author.

## Ethics statement

This animal study was reviewed and approved by Guangdong Laboratory Animal Monitoring Institute.

## Author contributions

TC: optimization of extraction process experiment, animal experiment, and article writing. DP: preliminary drug screening and experimental optimization. WZ: project advancement. SM and CY: literature search. XY: data processing of orthogonal experiment. JL: literature compilation. All authors contributed to the article and approved the submitted version.

## Footnotes

1
http://www.kegg.jp/kegg/pathway.html

## Abbreviations

Gym: *Gymnadenia conopsea* (L.) R. Br.
UA: uric acid
ROS: reactive oxygen species
BUN: blood urea nitrogen
CRE: creatinine
XOD: xanthine oxidase
SOD: superoxide dismutase
MDA: malondialdehyde
HUA: hyperuricemia
APL: allopurinol
PO: potassium oxonate
XSS: xanthine sodium salt
UHPLC: Ultra-high performance liquid chromatography
QE: Q-Exactive
MS: mass spectrometry
OPLS-DA: orthogonal projections to latent structures discriminant analysis
PCA: principal component analysis.
