# Supplementary material for: Optimization of the extraction process and metabonomics analysis of uric acid-reducing active substances from Gymnadenia R.Br. and its protective effect on hyperuricemia zebrafish
Source: Front Nutr. 2022 Dec 5;9:1054294. doi: 10.3389/fnut.2022.1054294 (PMC9760756; doi:10.3389/fnut.2022.1054294)
Supplement: Supplementary file 1 [file Data_Sheet_1.docx]

Supplementary materials：


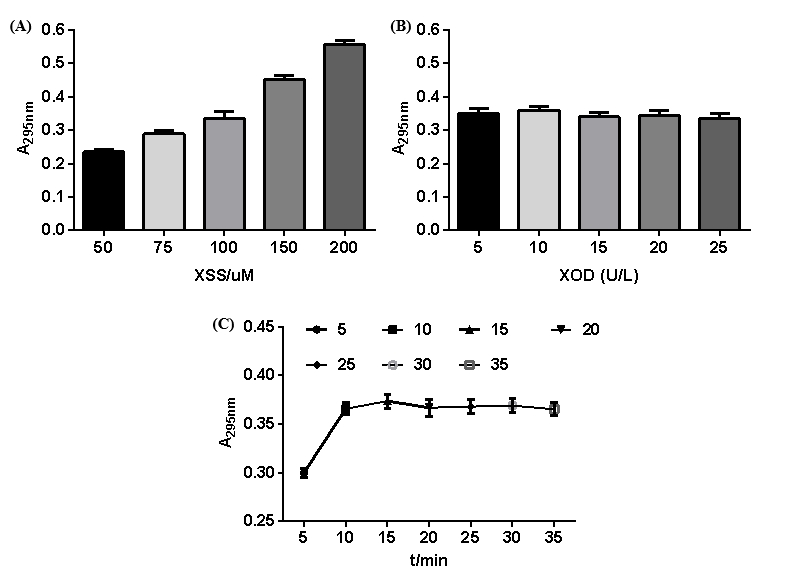


Supplementary Fig S1. Establishment of XOD inhibition model in vitro. (A): The effect of XSS concentration. (B): The effect of XOD concentration. (C): The effect of reaction time.


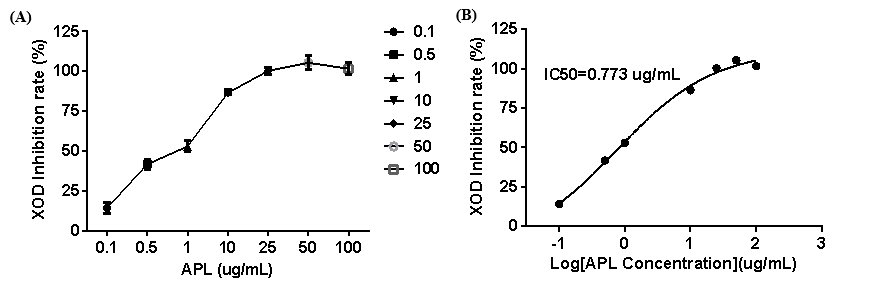


Supplementary Fig S2. (A): The effect of APL concentration on XOD inhibition rate. (B): APL half inhibitory concentration.

Supplementary Fig S3. The effect of solid-liquid ratio on extraction rate of ethanol extract of Gym.


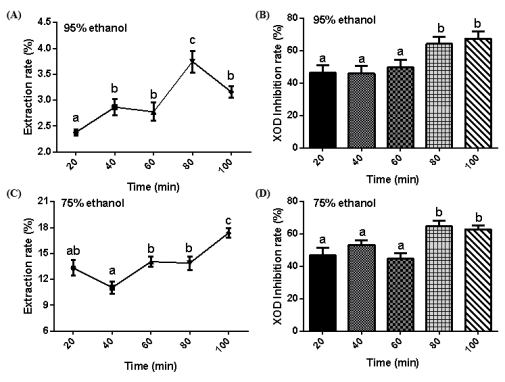


Supplementary Fig S4 The effect of ultrasonic time. (A)(C): The effect of ultrasonic time on extraction rate of ethanol extract of Gym((A):95%; (C):75%). (B)(D): The effect of ultrasonic time on XOD inhibition rate((B):95%; (D):75%).


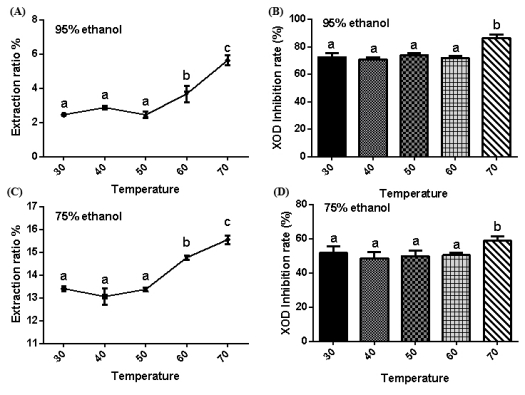


Supplementary Fig S5 The effect of extraction temperature. (A)(C): The effect of extraction temperature on extraction rate of ethanol extract of Gym((A):95%; (C):75%). (B)(D): The effect of extraction temperature on XOD inhibition rate((B):95%; (D):75%).

Supplementary Fig S6 The validation result of XOD inhibition of two ethanol extract in vitro


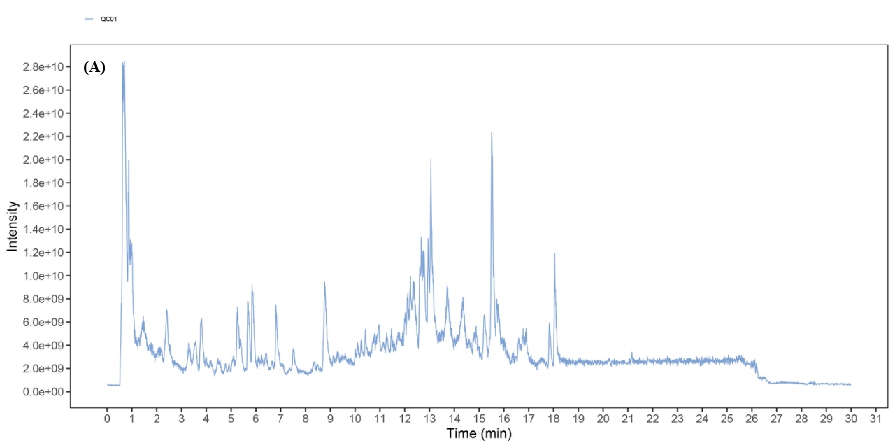


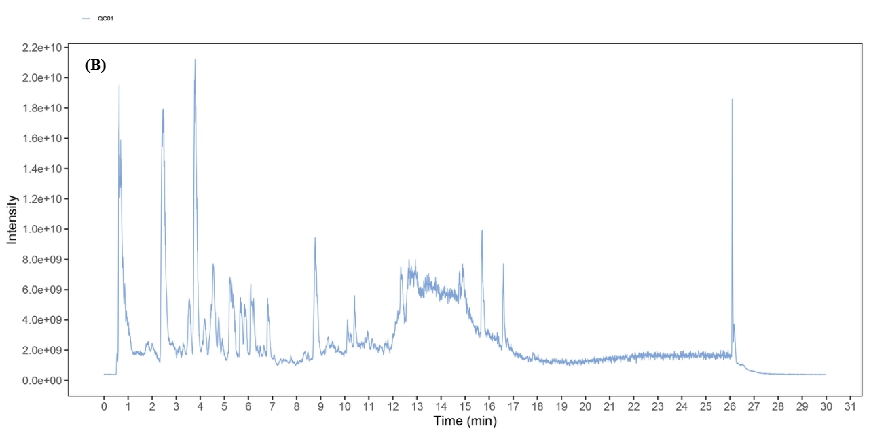


Supplementary Fig S7. The total ion current (TIC) of QC under positive and negative ions. (A): under positive; (B):under negative.


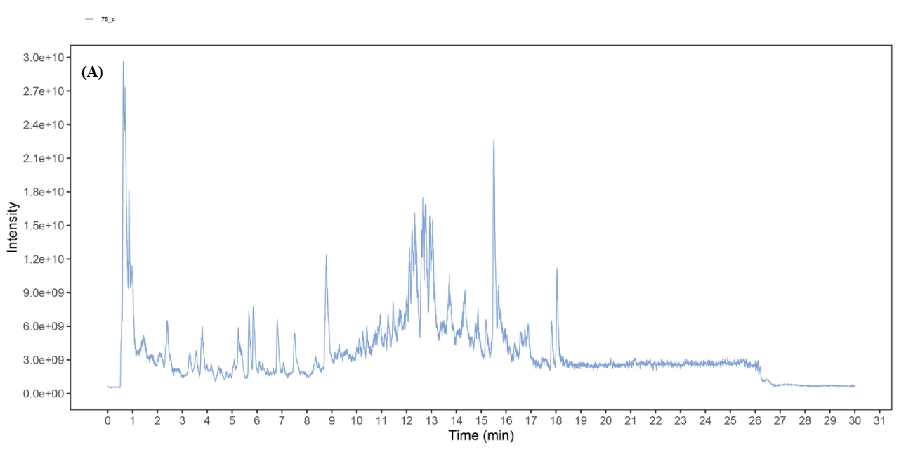


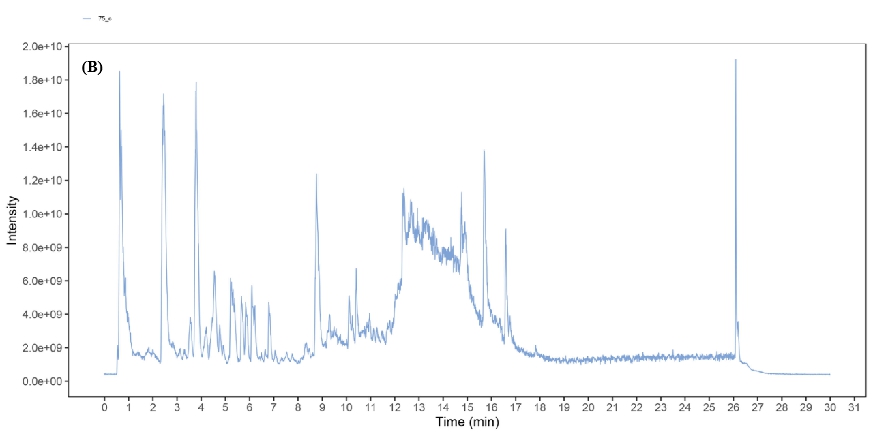


Supplementary Fig S8. The total ion current (TIC) of 95% ethanol extract of Gym under positive and negative ions. (A): under positive; (B):under negative.


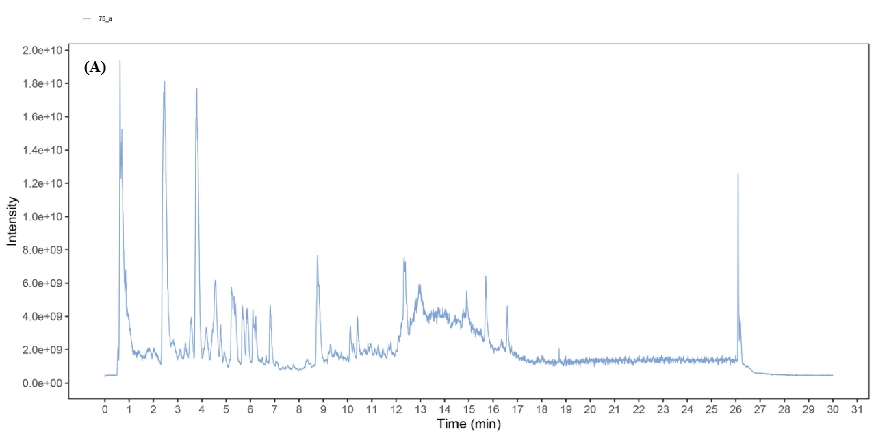


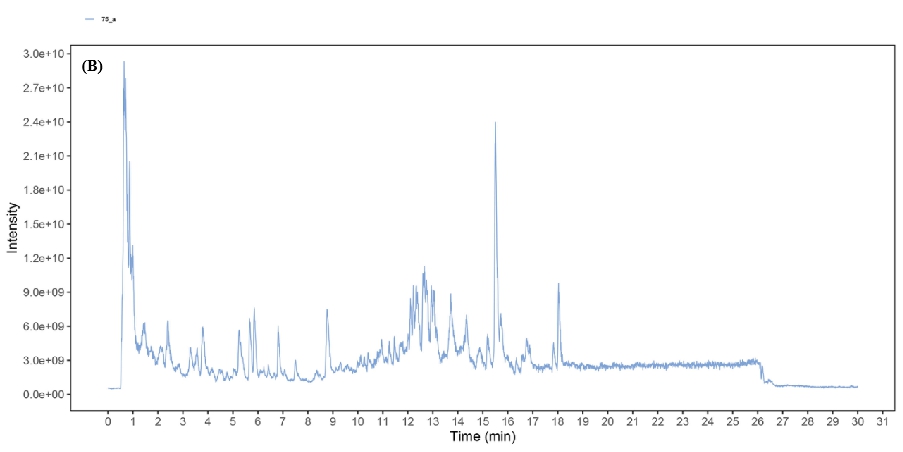


Supplementary Fig S9. The total ion current (TIC) of 75% ethanol extract of Gym under positive and negative ions. (A): under positive; (B):under negative.

Supplementary Table S1 Single factor experiment factors and levels

| Factor | Level | | | | |
| --- | --- | --- | --- | --- | --- |
|  | 1 | 2 | 3 | 4 | 5 |
| Liquid-solid ratio | 1:10 | 1:20 | 1:30 | 1:40 | 1:50 |
| Ethanol concentration | 55% | 65% | 75% | 85% | 95% |
| Ultrasonic power (W) | 200 | 300 | 350 | 400 | 500 |
| Ultrasonic time (min) | 20 | 40 | 60 | 80 | 100 |
| Extraction temperature (℃) | 30 | 40 | 50 | 60 | 70 |

Supplementary Table S2 Reaction system

| Group | PBS  (μL) | XOD  (μL) | XSS  (μL) | Sample  (μL) |
| --- | --- | --- | --- | --- |
| A0 | 50 | 50 | 100 | 0 |
| A1 | 100 | 0 | 100 | 0 |
| Ai | 0 | 50 | 100 | 50 |
| Aj | 50 | 0 | 100 | 50 |

Supplementary Table S3 Orthogonal experiment factors and levels

| Factor | Level | | |
| --- | --- | --- | --- |
|  | -1 | 0 | 1 |
| Ultrasonic power (W) | 200 | 350 | 500 |
| Liquid-solid ratio | 1:10 | 1:20 | 1:40 |
| Ultrasonic time (mins) | 20 | 40 | 80 |

Supplementary Table S4 The results of L9(3^4^) orthogonal test（n=3,95% ethanol extract)

| Run | A  Ultrasonic Power  W | B  Solid-liquid ratio  g/mL | C  Ultrasonic Time  min | Extraction rate % | Inhibition rate % |
| --- | --- | --- | --- | --- | --- |
| 1 | 200 | 1:10 | 20 | 2.87 | 63.94 |
| 2 | 200 | 1:20 | 40 | 3.98 | 62.77 |
| 3 | 200 | 1:40 | 80 | 4.53 | 64.47 |
| 4 | 350 | 1:10 | 20 | 2.62 | 64.94 |
| 5 | 350 | 1:20 | 80 | 3.26 | 59.26 |
| 6 | 350 | 1:40 | 40 | 3.52 | 54.80 |
| 7 | 500 | 1:10 | 40 | 2.99 | 64.51 |
| 8 | 500 | 1:20 | 20 | 3.42 | 65.08 |
| 9 | 500 | 1:40 | 80 | 4.32 | 84.02 |
| K1 | 191.18 | 193.39 | 193.96 |  |  |
| K2 | 179.00 | 187.11 | 182.08 |  |  |
| K3 | 213.61 | 203.29 | 207.75 |  |  |
| K1 | 63.73 | 64.46 | 64.65 |  |  |
| K2 | 59.67 | 62.37 | 60.69 |  |  |
| K3 | 71.20 | 67.76 | 69.25 |  |  |
| R | 11.53 | 5.39 | 8.56 |  |  |
|  | A>C>B |  |  |  |  |
|  | A3C3B3 |  |  |  |  |

Supplementary Table S5 The results of L9(3^4^) orthogonal test（n=3,75% ethanol extract)

| Run | A  Ultrasonic Power  W | B  Liquid-solid ratio g/mL | C  Ultrasonic Temperature  ℃ | Extraction rate % | Inhibition rate % |
| --- | --- | --- | --- | --- | --- |
| 1 | 200 | 1:10 | 20 | 10.22 | 47.56 |
| 2 | 200 | 1:20 | 40 | 12.70 | 59.88 |
| 3 | 200 | 1:40 | 80 | 14.99 | 71.05 |
| 4 | 350 | 1:10 | 20 | 10.06 | 49.77 |
| 5 | 350 | 1:20 | 80 | 13.52 | 58.32 |
| 6 | 350 | 1:40 | 40 | 14.46 | 59.39 |
| 7 | 500 | 1:10 | 40 | 10.17 | 58.99 |
| 8 | 500 | 1:20 | 20 | 12.02 | 68.06 |
| 9 | 500 | 1:40 | 80 | 14.68 | 76.84 |
| K1 | 178.49 | 156.32 | 165.39 |  |  |
| K2 | 167.48 | 178.26 | 178.26 |  |  |
| K3 | 203.89 | 207.28 | 206.21 |  |  |
| K1 | 59.50 | 52.11 | 55.13 |  |  |
| K2 | 55.83 | 59.42 | 59.42 |  |  |
| K3 | 67.96 | 69.09 | 68.74 |  |  |
| R | 12.13 | 16.98 | 13.61 |  |  |
|  | B>C>A |  |  |  |  |
|  | B3C3A3 |  |  |  |  |

Supplementary Table S6 Analysis of variance of average extraction rate (95% ethanol extract )

| Source of variance | Sum squares of deviations | df | variance | *F*-value | *P*-value |
| --- | --- | --- | --- | --- | --- |
| A | 0.6791 | 2 | 0.33954 | 5.01 | 0.166 |
| B | 0.9380 | 2 | 0.46902 | 6.92 | 0.126 |
| C | 0.1105 | 2 | 0.05525 | 0.81 | 0.551 |
| Error | 0.1356 | 2 | 0.06780 |  |  |

Supplementary Table S7 Analysis of variance of mean inhibition rate (95% ethanol extract )

| Source of variance | Sum squares of deviations | *df* | variance | *F*-value | *P*-value |
| --- | --- | --- | --- | --- | --- |
| A | 205.48 | 2 | 102.74 | 1.27 | 0.440 |
| B | 28.17 | 2 | 14.09 | 0.17 | 0.851 |
| C | 93.84 | 2 | 46.92 | 0.58 | 0.633 |
| Error | 161.53 | 2 | 80.77 |  |  |

Supplementary Table S8 Analysis of variance of average extraction rate (75% ethanol extract )

| Source of variance | Sum squares of deviations | *df* | variance | *F*-value | *P*-value |
| --- | --- | --- | --- | --- | --- |
| A | 0.2742 | 2 | 0.13708 | 8.80 | 0.102 |
| B | 12.5655 | 2 | 6.28274 | 403.14 | 0.002 |
| C | 0.9781 | 2 | 0.48907 | 31.38 | 0.031 |
| Error | 0.0312 | 2 | 0.01558 |  |  |

Supplementary Table S9 Analysis of variance of mean inhibition rate (75% ethanol extract )

| Source of variance | Sum squares of deviations | *df* | variance | *F*-value | *P*-value |
| --- | --- | --- | --- | --- | --- |
| A | 232.45 | 2 | 116.23 | 8.58 | 0.104 |
| B | 173.59 | 2 | 86.80 | 6.41 | 0.135 |
| C | 26.70 | 2 | 13.35 | 0.99 | 0.504 |
| Error | 27.10 | 2 | 13.55 |  |  |

Supplementary Table S10 (Annex 1 for the summary of differential metabolites)

| MS2 name | Class | VIP | P-VALUE | FOLD CHANGE | Status |
| --- | --- | --- | --- | --- | --- |
| Harpagoside | Phenylpropanoids | 1.6363 | 0.0492 | 0.0402 | Elevated |
| CITRATE | Organic acids and derivatives | 1.6430 | 0.0000 | 0.0480 | Elevated |
| 4-Methyl-6,7-dihydroxycoumarin | Phenylpropanoids | 1.5939 | 0.0022 | 0.0606 | Elevated |
| methyl (1R,4aS,9S,10S)-10-acetyloxy-5,9-dihydroxy-1,4a-dimethyl-2-oxo-7-propan-2-yl-10,10a-dihydro-9H-phenanthrene-1-carboxylate | Terpenoids | 1.6403 | 0.0001 | 0.0669 | Elevated |
| Arginine | Amino acid derivatives | 1.6389 | 0.0145 | 0.0744 | Elevated |
| Sibiricose A5 | Phenylpropanoids | 1.6325 | 0.0020 | 0.1513 | Elevated |
| asperuloside | Iridoids | 1.4414 | 0.0341 | 0.1521 | Elevated |
| Chelidonic acid (not validated) | Organic acids and derivatives | 1.5984 | 0.0001 | 0.1586 | Elevated |
| L-Tryptophan | Organoheterocyclic compounds | 1.4490 | 0.0070 | 0.1787 | Elevated |
| 7-(2-Hydroxy-2-propanyl)-1,4a-dimethyldecahydro-1-naphthalenol | Terpenoids | 1.5985 | 0.0392 | 0.1964 | Elevated |
| 3',5'-CYCLIC AMP | Alkaloids | 1.5280 | 0.0311 | 0.2163 | Elevated |
| L-Phenylalanine | Amino acid derivatives | 1.5561 | 0.0086 | 0.2688 | Elevated |
| Sakuranetin | Flavonoids | 1.5622 | 0.0089 | 0.2780 | Elevated |
| L-Cysteine | Amino acid derivatives | 1.5838 | 0.0018 | 0.3007 | Elevated |
| Rhaponticin | Phenols | 1.5885 | 0.0066 | 0.3088 | Elevated |
| 3-Hydroxybenzaldehyde | Phenols | 1.2797 | 0.0232 | 11.3471 | Reduced |
| Bullatine G | Terpenoids | 1.5968 | 0.0008 | 10.0673 | Reduced |
| 3-(2-Hydroxy-3,4-dimethoxyphenyl)-7-chromanol | Isoflavonoids | 1.6221 | 0.0486 | 8.0096 | Reduced |
| neoandrographolide | Terpenoids | 1.6299 | 0.0474 | 7.2579 | Reduced |
| Delphinidin | Tetraterpenes | 1.5588 | 0.0046 | 7.1991 | Reduced |
| Spiro[7H-cyclohepta[b]furan-7,2'(5'H)-furan]-2,5'(3H)-dione, octahydro-8-hydroxy-6,8-dimethyl-3-methylene-, (3aS,6S,7R,8aR)- | Terpenoids | 1.6158 | 0.0030 | 7.1551 | Reduced |
| (3aR,4R,6aR,8S,9aR,9bR)-4,8-Dihydroxy-3,6,9-tris(methylene)decahydroazuleno[4,5-b]furan-2(3H)-one | Terpenoids | 1.6162 | 0.0351 | 6.9701 | Reduced |
| Tulipinolide | Miscellaneous | 1.5837 | 0.0004 | 6.6363 | Reduced |
| Viridiflorine | Alkaloids | 1.3840 | 0.0042 | 6.6052 | Reduced |
| Lasiocarpine | Alkaloids | 1.5915 | 0.0064 | 6.0214 | Reduced |
| Oleic acid | Fatty acids | 1.5515 | 0.0035 | 5.5412 | Reduced |
| 3,4,5-trimethoxycinnamic acid | Phenylpropanoids | 1.5034 | 0.0388 | 5.4016 | Reduced |
| 2,4,6-trihydroxy-5-[1-(4-hydroxy-1,1,4,7-tetramethyl-1a,2,3,4a,5,6,7a,7b-octahydrocyclopropa[h]azulen-7-yl)-3-methylbutyl]benzene-1,3-dicarbaldehyde | Terpenoids | 1.4816 | 0.0116 | 5.0121 | Reduced |
| 3,5-Dimethoxy-4-hydroxybenzaldehyde | Phenols | 1.3244 | 0.0317 | 4.5220 | Reduced |
| Methyl Haematommate | Miscellaneous | 1.1155 | 0.0070 | 4.0312 | Reduced |
| MS2 name | Class | VIP | P-VALUE | FOLD CHANGE | Status |
| Harpagoside | Phenylpropanoids | 1.6363 | 0.0492 | 0.0402 | Elevated |
| CITRATE | Organic acids and derivatives | 1.6430 | 0.0000 | 0.0480 | Elevated |
| 4-Methyl-6,7-dihydroxycoumarin | Phenylpropanoids | 1.5939 | 0.0022 | 0.0606 | Elevated |
| methyl (1R,4aS,9S,10S)-10-acetyloxy-5,9-dihydroxy-1,4a-dimethyl-2-oxo-7-propan-2-yl-10,10a-dihydro-9H-phenanthrene-1-carboxylate | Terpenoids | 1.6403 | 0.0001 | 0.0669 | Elevated |
| Arginine | Amino acid derivatives | 1.6389 | 0.0145 | 0.0744 | Elevated |
| Sibiricose A5 | Phenylpropanoids | 1.6325 | 0.0020 | 0.1513 | Elevated |
| asperuloside | Iridoids | 1.4414 | 0.0341 | 0.1521 | Elevated |
| Chelidonic acid (not validated) | Organic acids and derivatives | 1.5984 | 0.0001 | 0.1586 | Elevated |
| L-Tryptophan | Organoheterocyclic compounds | 1.4490 | 0.0070 | 0.1787 | Elevated |
| 7-(2-Hydroxy-2-propanyl)-1,4a-dimethyldecahydro-1-naphthalenol | Terpenoids | 1.5985 | 0.0392 | 0.1964 | Elevated |
| 3',5'-CYCLIC AMP | Alkaloids | 1.5280 | 0.0311 | 0.2163 | Elevated |
| L-Phenylalanine | Amino acid derivatives | 1.5561 | 0.0086 | 0.2688 | Elevated |
| Sakuranetin | Flavonoids | 1.5622 | 0.0089 | 0.2780 | Elevated |
| L-Cysteine | Amino acid derivatives | 1.5838 | 0.0018 | 0.3007 | Elevated |
| Rhaponticin | Phenols | 1.5885 | 0.0066 | 0.3088 | Elevated |
| 3-Hydroxybenzaldehyde | Phenols | 1.2797 | 0.0232 | 11.3471 | Reduced |
| Bullatine G | Terpenoids | 1.5968 | 0.0008 | 10.0673 | Reduced |
| 3-(2-Hydroxy-3,4-dimethoxyphenyl)-7-chromanol | Isoflavonoids | 1.6221 | 0.0486 | 8.0096 | Reduced |
| neoandrographolide | Terpenoids | 1.6299 | 0.0474 | 7.2579 | Reduced |
| Delphinidin | Tetraterpenes | 1.5588 | 0.0046 | 7.1991 | Reduced |
| Spiro[7H-cyclohepta[b]furan-7,2'(5'H)-furan]-2,5'(3H)-dione, octahydro-8-hydroxy-6,8-dimethyl-3-methylene-, (3aS,6S,7R,8aR)- | Terpenoids | 1.6158 | 0.0030 | 7.1551 | Reduced |
| (3aR,4R,6aR,8S,9aR,9bR)-4,8-Dihydroxy-3,6,9-tris(methylene)decahydroazuleno[4,5-b]furan-2(3H)-one | Terpenoids | 1.6162 | 0.0351 | 6.9701 | Reduced |
| Tulipinolide | Miscellaneous | 1.5837 | 0.0004 | 6.6363 | Reduced |
| Viridiflorine | Alkaloids | 1.3840 | 0.0042 | 6.6052 | Reduced |
| Lasiocarpine | Alkaloids | 1.5915 | 0.0064 | 6.0214 | Reduced |
| Oleic acid | Fatty acids | 1.5515 | 0.0035 | 5.5412 | Reduced |
| 3,4,5-trimethoxycinnamic acid | Phenylpropanoids | 1.5034 | 0.0388 | 5.4016 | Reduced |
| 2,4,6-trihydroxy-5-[1-(4-hydroxy-1,1,4,7-tetramethyl-1a,2,3,4a,5,6,7a,7b-octahydrocyclopropa[h]azulen-7-yl)-3-methylbutyl]benzene-1,3-dicarbaldehyde | Terpenoids | 1.4816 | 0.0116 | 5.0121 | Reduced |
| 3,5-Dimethoxy-4-hydroxybenzaldehyde | Phenols | 1.3244 | 0.0317 | 4.5220 | Reduced |
| Methyl Haematommate | Miscellaneous | 1.1155 | 0.0070 | 4.0312 | Reduced |

Supplementary Table S11 KEGG (Annex 2 for metabolic pathway enrichment table)

| **Pathway** | **Description** | **# compounds (dem)** | **# compounds (all)** |
| --- | --- | --- | --- |
| ath01100 | Metabolic pathways - Arabidopsis thaliana (thale cress) | 18 | 50 |
| ath01110 | Biosynthesis of secondary metabolites - Arabidopsis thaliana (thale cress) | 18 | 37 |
| ath01230 | Biosynthesis of amino acids - Arabidopsis thaliana (thale cress) | 7 | 8 |
| ath00970 | Aminoacyl-tRNA biosynthesis - Arabidopsis thaliana (thale cress) | 6 | 6 |
| ath01240 | Biosynthesis of cofactors - Arabidopsis thaliana (thale cress) | 6 | 9 |
| ath02010 | ABC transporters - Arabidopsis thaliana (thale cress) | 5 | 11 |
| ath00460 | Cyanoamino acid metabolism - Arabidopsis thaliana (thale cress) | 4 | 4 |
| ath00944 | Flavone and flavonol biosynthesis - Arabidopsis thaliana (thale cress) | 4 | 5 |
| ath00966 | Glucosinolate biosynthesis - Arabidopsis thaliana (thale cress) | 4 | 4 |
| ath01210 | 2-Oxocarboxylic acid metabolism - Arabidopsis thaliana (thale cress) | 4 | 6 |
| ath00400 | Phenylalanine, tyrosine and tryptophan biosynthesis - Arabidopsis thaliana (thale cress) | 3 | 4 |
| ath00470 | D-Amino acid metabolism - Arabidopsis thaliana (thale cress) | 3 | 4 |
| ath00941 | Flavonoid biosynthesis - Arabidopsis thaliana (thale cress) | 3 | 5 |
| ath00999 | Biosynthesis of various plant secondary metabolites - Arabidopsis thaliana (thale cress) | 3 | 5 |
| ath00260 | Glycine, serine and threonine metabolism - Arabidopsis thaliana (thale cress) | 2 | 5 |
| ath00280 | Valine, leucine and isoleucine degradation - Arabidopsis thaliana (thale cress) | 2 | 2 |
| ath00290 | Valine, leucine and isoleucine biosynthesis - Arabidopsis thaliana (thale cress) | 2 | 3 |
| ath00770 | Pantothenate and CoA biosynthesis - Arabidopsis thaliana (thale cress) | 2 | 2 |
| ath00940 | Phenylpropanoid biosynthesis - Arabidopsis thaliana (thale cress) | 2 | 3 |
| ath00960 | Tropane, piperidine and pyridine alkaloid biosynthesis - Arabidopsis thaliana (thale cress) | 2 | 5 |
